# Spherical Harmonics Coefficients for Ligand-Based Virtual Screening of Cyclooxygenase Inhibitors

**DOI:** 10.1371/journal.pone.0021554

**Published:** 2011-07-27

**Authors:** Quan Wang, Kerstin Birod, Carlo Angioni, Sabine Grösch, Tim Geppert, Petra Schneider, Matthias Rupp, Gisbert Schneider

**Affiliations:** 1 Frankfurt Institute for Advanced Studies (FIAS), Goethe University, Frankfurt, Germany; 2 Institute for Clinical Pharmacology, Goethe University, Frankfurt, Germany; 3 Institute of Pharmaceutical Sciences, Swiss Federal Institute of Technology (ETH), Zürich, Switzerland; 4 Machine Learning Group, Technical University, Berlin, Germany; Charité-Universitätsmedizin Berlin, Germany

## Abstract

**Background:**

Molecular descriptors are essential for many applications in computational chemistry, such as ligand-based similarity searching. Spherical harmonics have previously been suggested as comprehensive descriptors of molecular structure and properties. We investigate a spherical harmonics descriptor for shape-based virtual screening.

**Methodology/Principal Findings:**

We introduce and validate a partially rotation-invariant three-dimensional molecular shape descriptor based on the norm of spherical harmonics expansion coefficients. Using this molecular representation, we parameterize molecular surfaces, i.e., isosurfaces of spatial molecular property distributions. We validate the shape descriptor in a comprehensive retrospective virtual screening experiment. In a prospective study, we virtually screen a large compound library for cyclooxygenase inhibitors, using a self-organizing map as a pre-filter and the shape descriptor for candidate prioritization.

**Conclusions/Significance:**

12 compounds were tested *in vitro* for direct enzyme inhibition and in a whole blood assay. Active compounds containing a triazole scaffold were identified as direct cyclooxygenase-1 inhibitors. This outcome corroborates the usefulness of spherical harmonics for representation of molecular shape in virtual screening of large compound collections. The combination of pharmacophore and shape-based filtering of screening candidates proved to be a straightforward approach to finding novel bioactive chemotypes with minimal experimental effort.

## Introduction

Ligand-based virtual screening [1], [2], quantitative structure-property and structure-activity relationships [3], [4], and other concepts in computational medicinal chemistry are based on the similarity principle [5], which states that (structurally) similar compounds generally exhibit similar properties. Such methods require quantitative representations of molecules, usually in the form of chemical descriptors, i. e., computable numerical attributes in vector form [6].

Numerous molecular 3D-descriptors and alignment methods have been proposed. Examples include CoMFA (comparative molecular field analysis) [7], Randic molecular profiles [8], 3D-MoRSE code (3D-molecule representation of structures based on electron diffraction) [9], invariant moments and radial scanning and integration [10], radial distribution function descriptors [11], WHIM (weighted holistic invariant molecular descriptors) [12], length-to-breadth ratios [13], USR (ultrafast shape recognition, based on statistical moments) [14], ROCS (rapid overlay of chemical structures, based on Gaussian densities) [15], VolSurf (volumes and surfaces of 3D molecular fields) [16], GETAWAY (geometry, topology, and atom weights assembly) [17], and shrink-wrap surfaces [18], to name just a few prominent representatives.

In computer graphics, several methods exist for the more general problem of comparing arbitrary 3D objects [19], [20], including distribution-based shape histograms [21], the D2 shape descriptor [22], and, the scaling index method [23]; the view-based methods of extended Gaussian images [24], and the light field descriptor [25]; the surface decomposition-based methods of Zernike moments [26], REXT (radialized spherical extent function) [27], and spherical harmonics descriptors [28].

Spherical harmonics have been used in cheminformatics as a global feature-based parametrization method of molecular shape [28]–[38]. Their attractive properties with regard to rotations make them an intuitive and convenient choice as basis functions when searching in a rotational space [31]. A review article by Venkatraman et al. [38] highlights applications of spherical harmonics to protein structure comparison, ligand binding site similarity, protein-protein docking, and virtual screening. Jakobi et al. [37] use spherical harmonics in their ParaFrag approach to derive 3D pharmacophores of molecular fragments. Recently, Ritchie and co-workers have applied the ParaSurf and ParaFit methodologies [32], [33] (Cepos InSilico Ltd., Erlangen, Germany) in a virtual screening study on the directory of useful decoys (DUD) data set [39], which motivates 3D shape-property combinations specifically for flexible ligands [40]. The DUD data set was also used in a comparative analysis of the performance of various shape descriptors alone and in combination with property and pharmacophore features [41]. See the section on related methods for further discussion of spherical harmonics approaches.

In this work, we introduce a partially rotation-invariant descriptor of molecular shape based on spherical harmonics decomposition coefficients. The idea is to decompose the molecular surface using spherical harmonics and to use the norm of the decomposition coefficients as a description of molecular shape. In this, we take advantage of the fact that the norm of the coefficients does not change under rotation around the 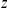-axis, which we align to the primary axis of the molecule. We retrospectively evaluate our descriptor, and prospectively apply it to screen for novel inhibitors of the enzymes cyclooxygenase-1 (COX-1) and cyclooxygenase-2 (COX-2). Particular focus is on the practical application of the virtual screening technique as an evaluation of its actual suitability for early-phase drug discovery.

## Materials and Methods

### Spherical harmonics

**Figure 1:**
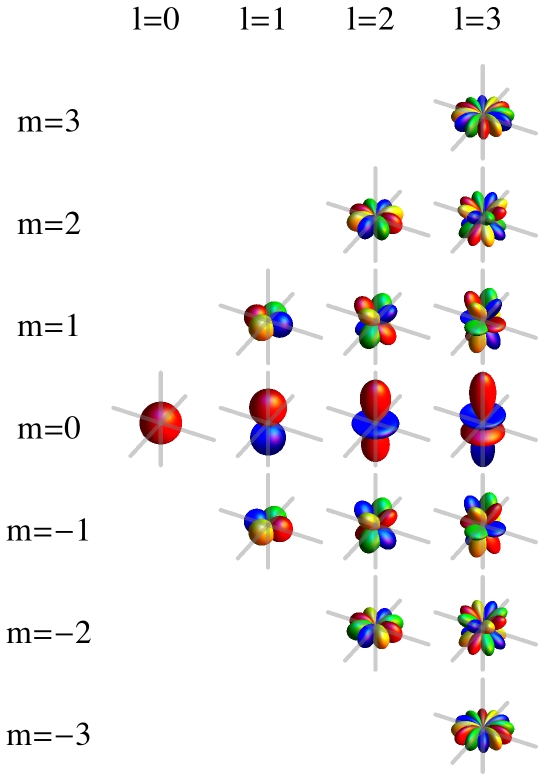
Spherical harmonics by order 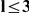 (columns, left to right) and degree 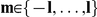 (rows, bottom to top). Shown are negative real (blue), positive real (red), negative imaginary (green), and positive imaginary (yellow) parts of 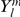.

Let 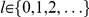, let 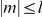, let 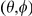 indicate spherical coordinates, and let 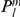 denote the Legendre polynomials [42]. The spherical harmonics [43] 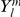 of order 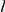 (frequency, angular quantum number) and degree 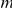 (azimuthal quantum number),
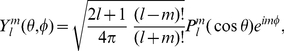
(1)form an orthonormal (with respect to integration over the unit sphere) and complete set of basis functions (Fig. 1). They are solutions to Laplace's differential equation 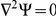 in spherical coordinates [44].

Any square-integrable spherical function 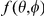 can be decomposed as
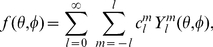
(2)with complex coefficients 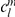. The spherical harmonics decomposition can be viewed as a generalization of the Fourier decomposition to three dimensions [45].

The coefficients 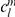 of an harmonic expansion can be found using the orthonormality property. Multiplying each side of Eq. 2 by the complex conjugate 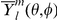 and integrating over the sphere yields
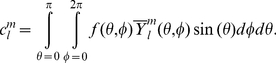
(3)Small values of 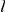 correspond to low frequencies, and describe the overall low-resolution shape; higher values of 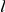 add finer, high-frequency detail. The coefficients are unique, and can therefore be used as feature vectors for shape description.

Rotation of a molecule (its shape function 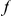) changes the coefficients. A conventional solution is to define a canonical orientation of the molecule. For the purpose of shape comparisons, this implies an alignment of the compared molecules, with all associated problems and computational requirements. As an alternative, we use a partial orientation in conjunction with certain rotational invariance properties of the coefficients.

### Descriptor definition

Let 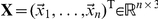 denote the Cartesian coordinates of points 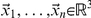 sampled from a molecular surface. We assume that the surface is “star-like” (single-valued) in the sense that rays radiating outward from the molecule's origin intersect the surface only once (this is more of an issue for proteins; as argued elsewhere [35], small molecules are little, if at all, affected). Let 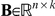 denote the spherical harmonics basis functions 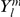 evaluated at 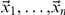, 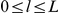, 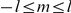, with 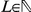 the maximum order used and 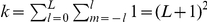 the number of basis functions. The sampled molecular surface 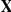 can be reconstructed using a matrix 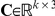 of coefficients as 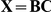. The coefficient matrix is given by 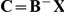, where 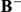 denotes the *pseudo*-inverse [46] of 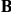.

#### Lemma

The 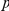-norm of the rows of 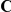 does not change under rotation around the 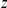-axis (polar axis, change in 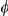).

*Proof* It is sufficient to consider a single coefficient, i.e., 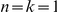, and 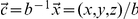. Here, 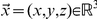 is a sampled surface point, 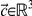 are coefficients, and 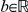 is the spherical harmonics basis function 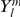. From Eq. 1, it is clear that if 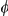 changes, only the part 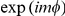 of the spherical harmonic basis function 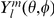 changes, while the rest of 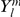 stays constant. Thus, 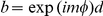 for some constant 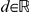 depending only on 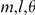, but not on 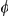. Since 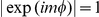, and thus 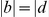,
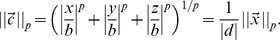
(4)After a rotation around the 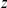-axis (a change in 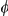), the same holds for the rotated point 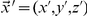 and its coefficient 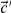, i.e., 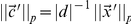. Since the rotation matrix is unitary, 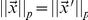, and it follows that 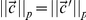.

Our spherical harmonics descriptor is the 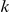-component vector
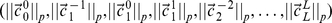
of the norms of the coefficients. It is a description of molecular shape that is invariant to rotations around the 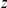-axis.

Before spherical harmonics decomposition, we place molecules into a common frame of reference by translating their center of gravity to the coordinate system origin and by aligning their first principle component (the direction of maximum variance as given by principle component analysis [47]) with the 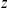-axis. In other words, we align molecules according to their longest spatial extent, and then apply our descriptor which is invariant to rotations around the 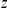-axis.

### Descriptor computation

**Figure 2:**
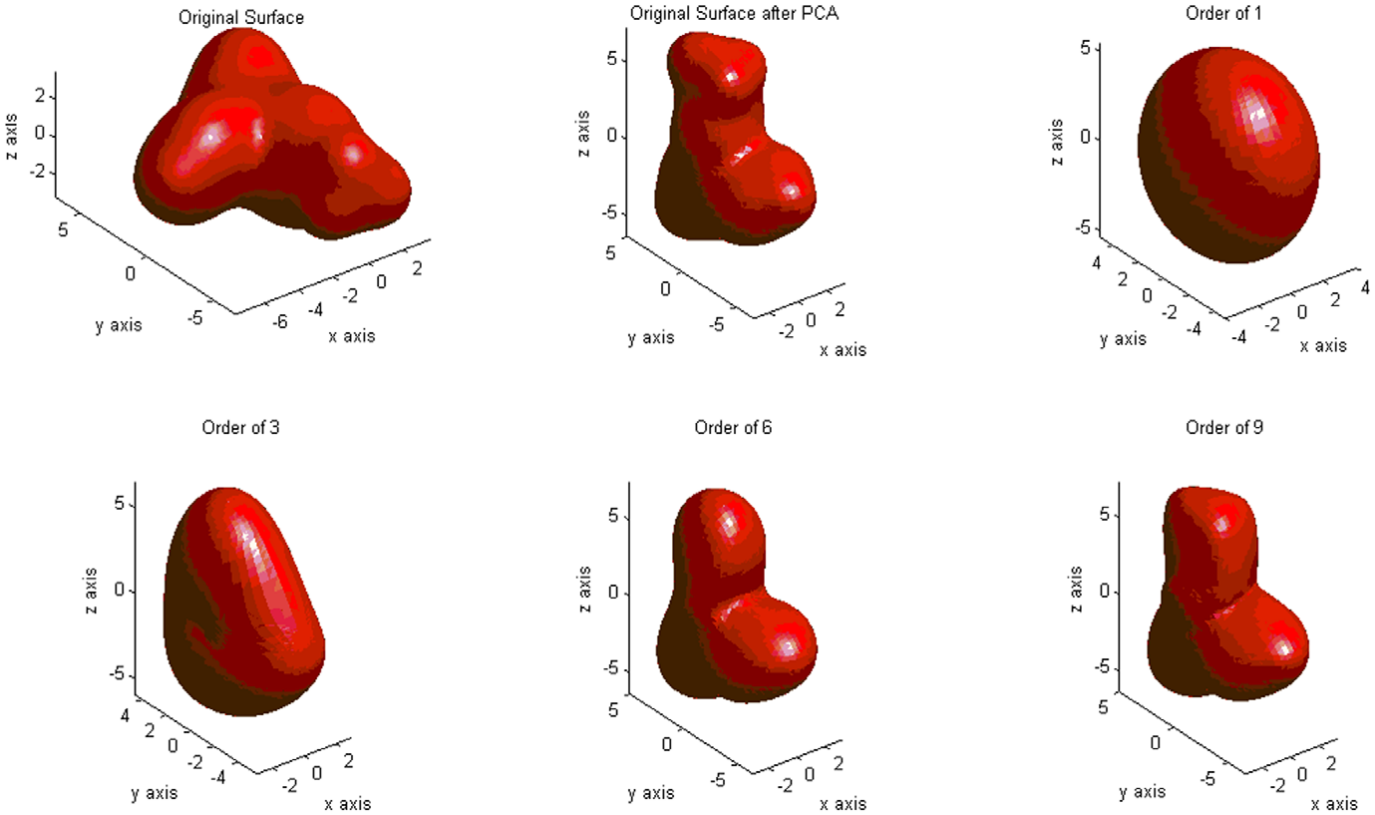
Surface reconstruction using spherical harmonics. Shown are the original surface (top left), the surface after alignment to the 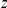-axis (top middle), and reconstructions using spherical harmonics of order 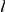 up to 1 (top right), 3 (bottom left), 6 (bottom middle), and 9 (bottom right).

**Figure 3:**
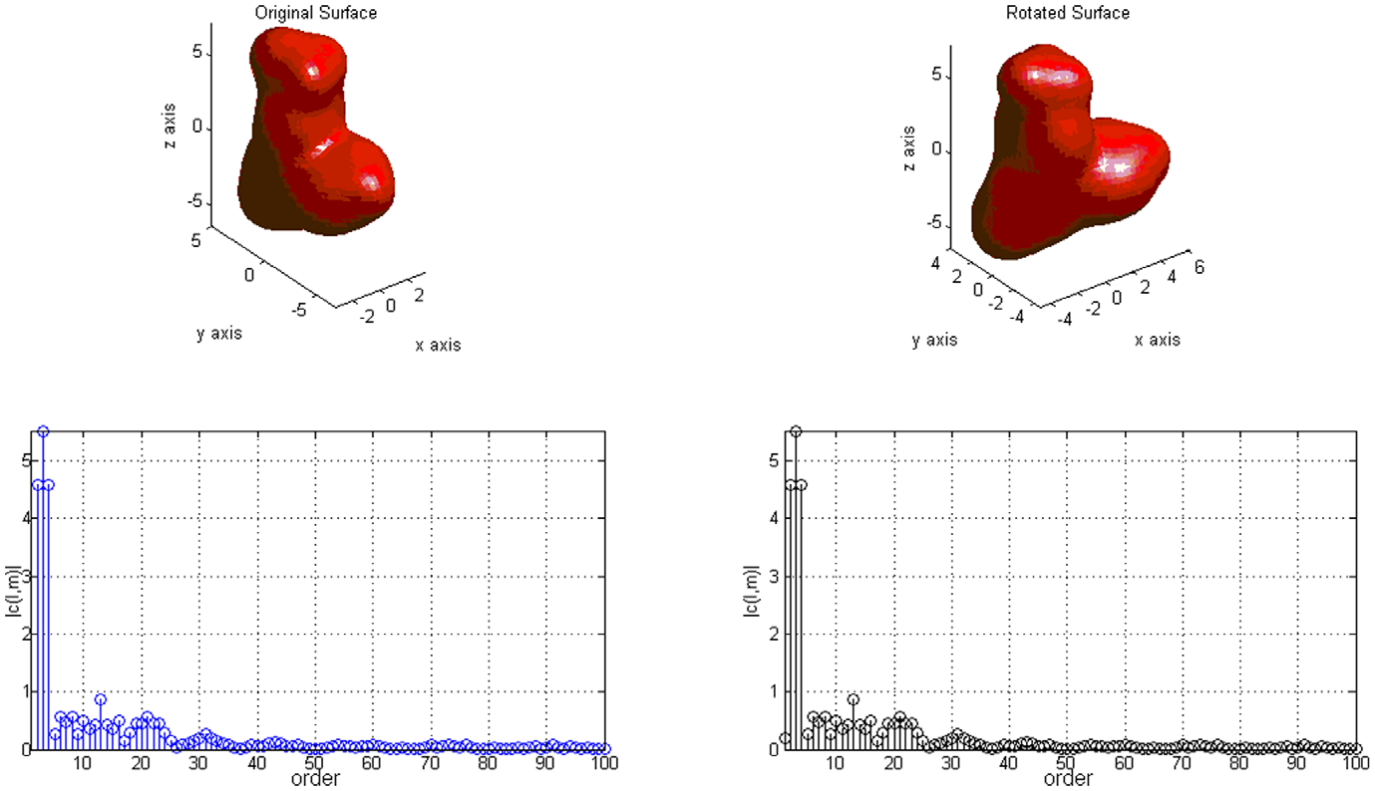
Spherical harmonics decomposition coefficients 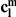 of a molecular surface for 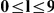. The original (top left) and the rotated (top right) surfaces yield coefficients with identical norm (bottom), up to numerical noise (differences were below 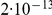).

Gaussian contact surfaces [48] of all compounds were computed using MOE (Molecular Operating Environment, version 2009.10, Chemical Computing Group Inc., Montreal, Canada, www.chemcomp.com). Spherical harmonics decomposition was then carried out on the vertices of these surfaces, giving approximate coefficients [49]. To limit computational expense, we truncated spherical harmonics expansions after order 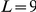. The resulting 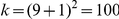 decomposition coefficients were sufficient to represent fine molecular detail and approximately reconstruct the original molecular surfaces (Fig. 2). The partial rotational invariance of the coefficient norms 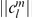 is demonstrated in Fig. 3. Computation was done in Matlab (The MathWorks, version R2007a, www.mathworks.com), partly based on code by Dr. Andrew Hanna (University of East Anglia, United Kingdom, www.cmp.uea.ac.uk/~aih ). Average computing time was 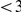 seconds per compound, which is acceptable for medium-sized libraries but will require speed-up for high-throughput virtual screening.

### Related methods

Spherical harmonics have been widely used in cheminformatics as a global feature-based parametrization method of molecular shape [28]–[38]. Most current approaches, including ours, use the center of gravity as the center of the spherical harmonics decomposition. Molecular surface sampling can be done by sampling iso-probability surfaces of molecular property densities. One aspect in which methods differ is the way they deal with rotations in 3D space.

Ritchie and Kemp [31] apply the rotational property of spherical harmonics (a rotation of the surface can be simulated by rotating the expansion coefficients) to maximize the pairwise superposition of two molecules. The software ParaSurf superposes molecules using a brute-force rotational search over the three Euler rotation angles [50]. In a recent publication, Cai et al. [36] use a similar approach to obtain the minimal root-mean-square distance between a ligand molecule and a target protein. In these related studies, molecular surfaces were rotated by transforming their expansion coefficients.

Standard orientation of compounds prior to spherical harmonics decomposition was proposed by Morris et al. [34]. Their work registered molecules and binding pockets in a standard frame by translating their center of mass to the coordinate origin and aligning their variance-covariance matrix to the axes of the coordinate system. They then use the coefficients of a real spherical harmonics expansion to describe and compare the molecular shape of binding pockets and ligands. This approach aligns molecules to minimize rotation-dependent differences in the coefficients.

Rotation-invariant spherical harmonics descriptors were applied by Kazhdan et al. [28] and Mavridis et al. [35], [51], using the fact that expansion coefficients of the same order 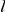 transform among themselves to construct rotationally invariant spherical harmonics coefficients 
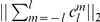
. In their approach, coefficients of the same order 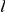 are binned together, thereby losing information contained in the individual degrees 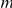, but gaining complete rotational invariance.

In this work, we combine partial orientation of the molecules with the magnitude of the expansion coefficients as a partially rotation-invariant shape descriptor. Our proposed descriptor retains more information than the spherical harmonics descriptors by Kazhdan et al. [28] and Mavridis et al. [35], [51] in the sense that coefficients within the same order are not summed up, but kept. Compared with standard orientation methods, our descriptor is potentially less susceptible to problems in the orientation step than most others because only the first (and most stable) principle component is used for orientation.

### Retrospective evaluation

For retrospective validation, we ranked the compounds in a database according to their similarity to a reference compound, as measured by Euclidean distance and our descriptor. Two conceptually different collections of reference data were used, the DUD data set (release 2, from http://dud.docking.org/r2 , unmodified data) [39], and the COBRA data set (version 10.3, 11 244 compounds annotated with activity on a total of 677 individual macromolecular targets) [52]. COBRA 10.3 contains 168 COX-2 inhibitors.

Gaussian contact surfaces were generated with the MOE 2009.10 (Molecular Operating Environment, Chemical Computing Group Inc., Montreal, Canada, www.chemcomp.com ) GaussianSurface function, with parameter pos set to ‘aPos a’, rad ‘dock_aRadius a’, nearpos ‘aPos a’, neardist ‘5’, maxMb ‘1’, and fuzzy ‘0’. All other parameters were kept at their default values. Virtual screening experiments in COBRA were carried out using a single conformation generated by CORINA (version 2007, Molecular Networks GmbH, Erlangen, Germany, www.molecular-networks.com ).

We used the selective COX-2 inhibitor SC-558 and the non-selective inhibitor indomethacin as queries for ligand-based similarity searching, with the conformations extracted from the crystal structure (protein data bank [53] identifiers (PDB ID) 6cox [54] and 4cox [54]). Enrichment factors [55], receiver operating characteristic curves (ROC curves [56]), and the area under these curves (ROC AUC) were used as performance measures.

### Prospective virtual screening

We screened the ChemBridge compound pool (457 226 compounds, ChemBridge Corp., San Diego, USA, www.chembridge.com) for potential COX ligands using a single CORINA conformer query as in the retrospective screening. The database was preprocessed using the “washing” procedure in MOE (protonation of strong bases and de-protonation of strong acids; all other parameters were kept at their default values).

To reduce computational effort and allow for pharmacophore feature-based compound ranking, the screening compound pool was pre-filtered using a self-organizing map (SOM [57]) trained on the ChemBridge collection and 275 COX-1 and COX-2 inhibitors from the COBRA database. SOM topology was toroidal with 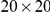 neurons (1 200 molecules per neuron on average); compounds were represented using the 150-dimensional CATS2D topological pharmacophore descriptor [58], [59] and compared using the Manhattan distance. The initial width of the Gaussian neighborhood function was set to 5; training was terminated after 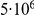 steps (using each compound 10 times on average). We used the MOLMAP software tool for SOM generation [60].

**Figure 4:**
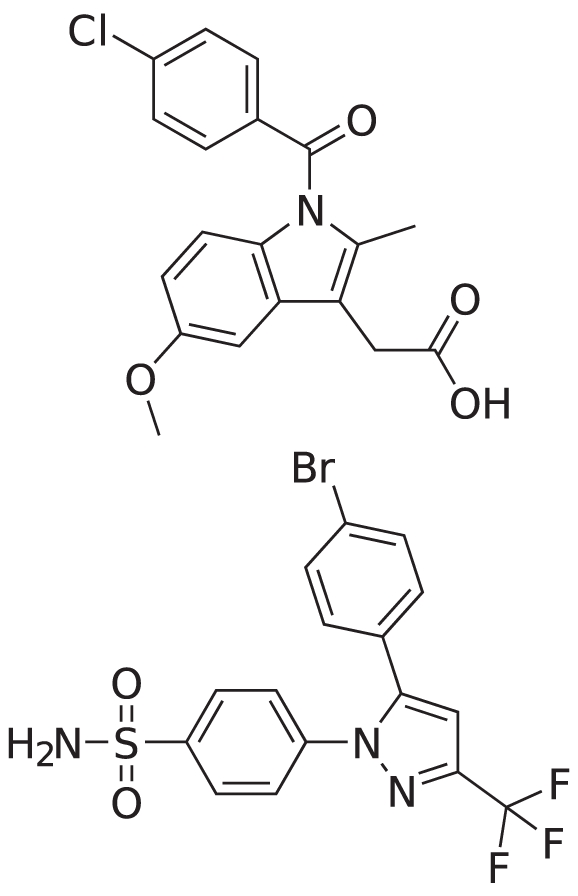
Reference COX inhibitors used for prospective screening with the shape descriptor. Indomethacin (top, PDB ID 4cox), a non-selective COX inhibitor, and, SC-558 (bottom, PDB ID 6cox), a selective COX-2 inhibitor.

After pre-filtering, 21 950 compounds of the ChemBridge database that were similar to the COX inhibitors from the COBRA database were retained for virtual screening using our spherical harmonics shape descriptor. Two potent COX inhibitors served as reference molecules (queries; Fig. 4). All parameters were set to the values used in retrospective virtual screening. The spherical harmonics descriptor was calculated for the 21 950 retained molecules and for the two reference molecules.

### Enzyme inhibition assay

Inhibition of COX-1 (ovine) and COX-2 (human recombinant) activity was measured using a COX inhibitor screening assay kit (Cayman Chemicals, Ann Arbor, MI, USA, www.caymanchem.com ), according to the manufacturer's protocol. SC-560, a selective COX-1 inhibitor, and celecoxib, a selective COX-2 inhibitor, served as positive controls. The COX inhibitor screening assay directly measures the amount of prostaglandins 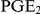, 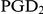 and 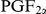 produced by 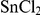 reduction of COX-derived 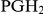. In addition to this protocol, the amounts of prostaglandins were quantified by LC-MS/MS analysis as described previously [61].

### Whole blood assay

#### COX-1 whole blood assay

One-milliliter heparinized human blood samples were incubated with 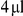 test substance (in DMSO) or 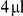 DMSO (control) for 10 min at 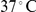. After this, thrombocyte aggregation was stimulated by addition of calcium ionophore A23187 (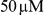) for 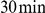 at 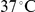. Plasma was separated by centrifugation for 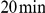 at 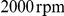, 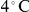 and kept at 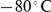 until assayed for 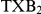 by LC-MSMS (see below).

#### COX-2 whole blood assay

For the determination of COX-2 activity, 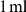 of heparinized human blood was incubated at 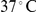 with 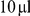 of acetylsalicylic acid (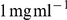 in PBS), 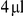 DMSO or 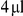 inhibitor (in DMSO) for 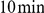. After this, 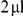 of LPS (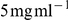 in DMSO) was added and incubated for 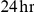 at 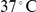. The reaction was terminated by quickly chilling on ice. Plasma was separated by centrifuging (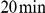, 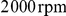, 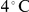), stored at 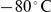 until analysis of prostaglandins by LC-MS/MS within two weeks.

#### Extraction procedure


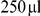
 plasma was incubated with 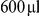 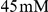 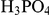, 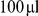 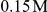 EDTA, 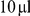 BHT (butylated hydroxytoluene, 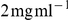), 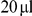 MeOH, 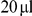 internal standard 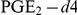 (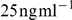), 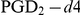 (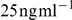), 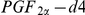 (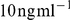), 6k 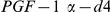 (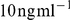), 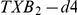 (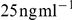) for 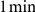, and passed through a ABS ELUT-Nexus cartridge (Varian, Darmstadt, Germany) preconditioned with methanol (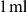), followed by distilled water (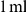). The cartridge was washed with distilled water (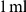) and 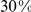 MeOH (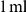). 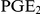, 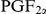, 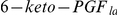 and 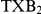 were eluted with hexane-ethylacetate-isopropranolol (30∶65∶5, v/v, 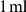). After vaporating the solvent under nitrogen atmosphere, the residue was reconstituted in 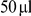 acetonitrile / formic acid. 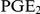 concentrations were quantified by means of a validated LC-MS/MS assay described previously [61]. The lower limit of quantification was 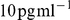.

## Results and Discussion

We validated our spherical harmonics (SpH) descriptor in a retrospective setting (statistical validation on known data), and in a prospective study to obtain biochemical confirmation of our model.

### Retrospective evaluation

As a first analysis, we used the DUD compound collection for a preliminary comparison of selected shape- and structure-based virtual screening methods. ROC AUC [62] values were computed for each of the methods compared. ROC AUC values lie in the interval 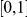, with values closer to 1 indicating higher enrichment of actives in a ranked list of compounds. The analysis was limited to the original COX-2 data from DUD (426 actives, 13 289 decoys). We did not perform exhaustive comparative analyses of virtual screening performance or focus on ‘early recognition’ of actives [63], [64], as the primary purpose of this study was to determine whether our SpH descriptor might be a useful shape-based filtering criterion for COX inhibitors. Retrospective screening was restricted to COX-2, our original target.

Table 1 summarizes the results obtained for CATS2D (topological pharmacophore descriptor [58]), LIQUID (three-dimensional pharmacophore descriptor using Gaussian feature points; v1: hydrogen-bond donors, hydrogen-bond acceptors, lipophilic [65]; v2: additional aromatic, positive and negative charge features (manuscript in preparation)), PRPS (Gaussian pseudoreceptor model [66], [67]), ShaEP (field-based subgraph matching [68]), and ROCS (Gaussian shape model [15], [69]). For the DUD COX-2 data, ROC AUC values indicate better than random performance for all methods. SpH yielded an average of 0.86, which compares to Ritchie's ParaFit spherical harmonics descriptor (note that the ParaFit ROC AUC value is not given in the original publication; we estimated it from graphical material provided in the article's supplementary material [41]). Among the tested methods, SpH performed best for the selective COX-2 inhibitor SC-558 (Fig. 4) yielding a ROC AUC = 0.91. Notably, high values were also obtained for indomethacin (Fig. 4), a non-selective COX inhibitor (COX-1 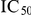 = 18 nM; COX-2 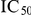 = 26 nM) [70]. Apparently, only the PRPS pseudoreceptor model distinguished between the selective (ROC AUC = 0.83) and the non-selective (ROC AUC = 0.15) query.

**Table 1:** Results (ROC AUC) of retrospective virtual screening of DUD data set for COX-2 inhibitors.

	Single query		DUD COX-2	
Method	SC558	Indomethacin	average 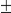 std.dev.	
CATS2D	0.76	0.49	0.63±	0.14
LIQUID v.1	0.59	0.60	0.58±	0.10
LIQUID v.2	0.80	0.61	0.74±	0.12
PRPS	0.83	0.15	0.76±	0.19
SpH	0.91	0.84	0.86±	0.12
ShaEP	0.89	0.79	0.61±	0.03^a^
ROCS	n.d.	n.d.	0.68^a^	

a Values reproduced from the original study by Vainio *et al.* [68], with ShaEP and ROCS run in ‘shape-only’ mode.

DUD COX-2 data: 426 actives, 13 289 decoys. Query SC558 has PDB ID 6cox; indomethacin has PDB ID 4cox. n.d. = not determined.

**Figure 5:**
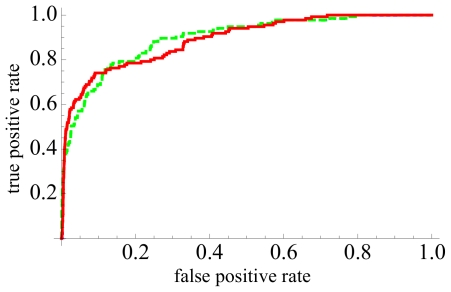
Receiver operating characteristic (ROC) curves for virtual screening by ranking against the COX-2 ligand SC-558 (PDB ID 6cox). Shown are curves for shapelets (solid red line), and spherical harmonics descriptor (dashed green line).

In contrast to DUD (unmodified data), the COBRA database contains only druglike bioactive compounds. Ranking of the COBRA database with SC-558 as query resulted in an enrichment factor (computed for the first percentile) of 23. We compared this result to those obtained by the shapelets [71] method from our group, using the same version of the COBRA database and the same reference structure. The shapelets shape-only virtual screening method achieved a comparable enrichment factor of 24. ROC curves are presented in Fig. 5 (numbers for shapelets refer to COBRA version 8.4 containing 8 311 compounds including 136 COX-2 inhibitors).

In summary, our spherical harmonics coefficients-based approach SpH achieves notable enrichment of actives and seems suitable for COX-2 inhibitor retrieval. This outcome is in agreement with the study of shape-based virtual screening approaches by Ritchie et al. [41], who report high hit rates for COX-2 using shape descriptors. We conclude that spherical harmonics-based decomposition of molecular shape captures structural features that are relevant for virtual screening. Due to the limited number of published prospective applications [72], it seems premature to render any conclusion regarding certain implementation preferences or ‘best-in-class’ spherical harmonics methods. To further assess our SpH approach, we performed a prospective study using SpH in a virtual screening cascade with the aim to identify new COX inhibitors.

### Prospective virtual screening

#### Virtual screening

**Figure 6:**
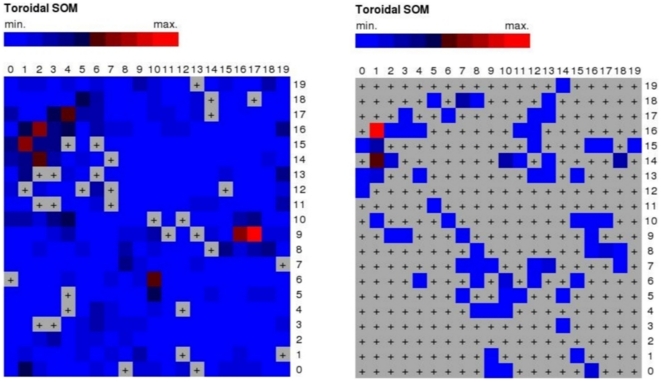
Self-organizing map projection of the ChemBridge database in CATS topological pharmacophore space, using a 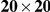 toroidal grid. Colors correspond to the number of compounds clustered, separately scaled for each plot, with 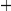 indicating empty neurons. The left panel presents the distribution of the 457 226 compounds from the ChemBridge database, the right panel shows the 275 COX ligands from the COBRA database.

We used a SOM to pre-select potential COX inhibitors from the screening compound pool. The SOM (Fig. 6) of COX activity islands contains six neurons with more than three ligands (neurons (1,16), (1,14), (1,15), (7,18), (18,14), (10,14) with 49, 25, 15, 14, 12, 11 ligands, respectively). We selected all compounds from the ChemBridge database contained in these neurons, 21 950 in total (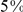 of the pool).

**Figure 7:**
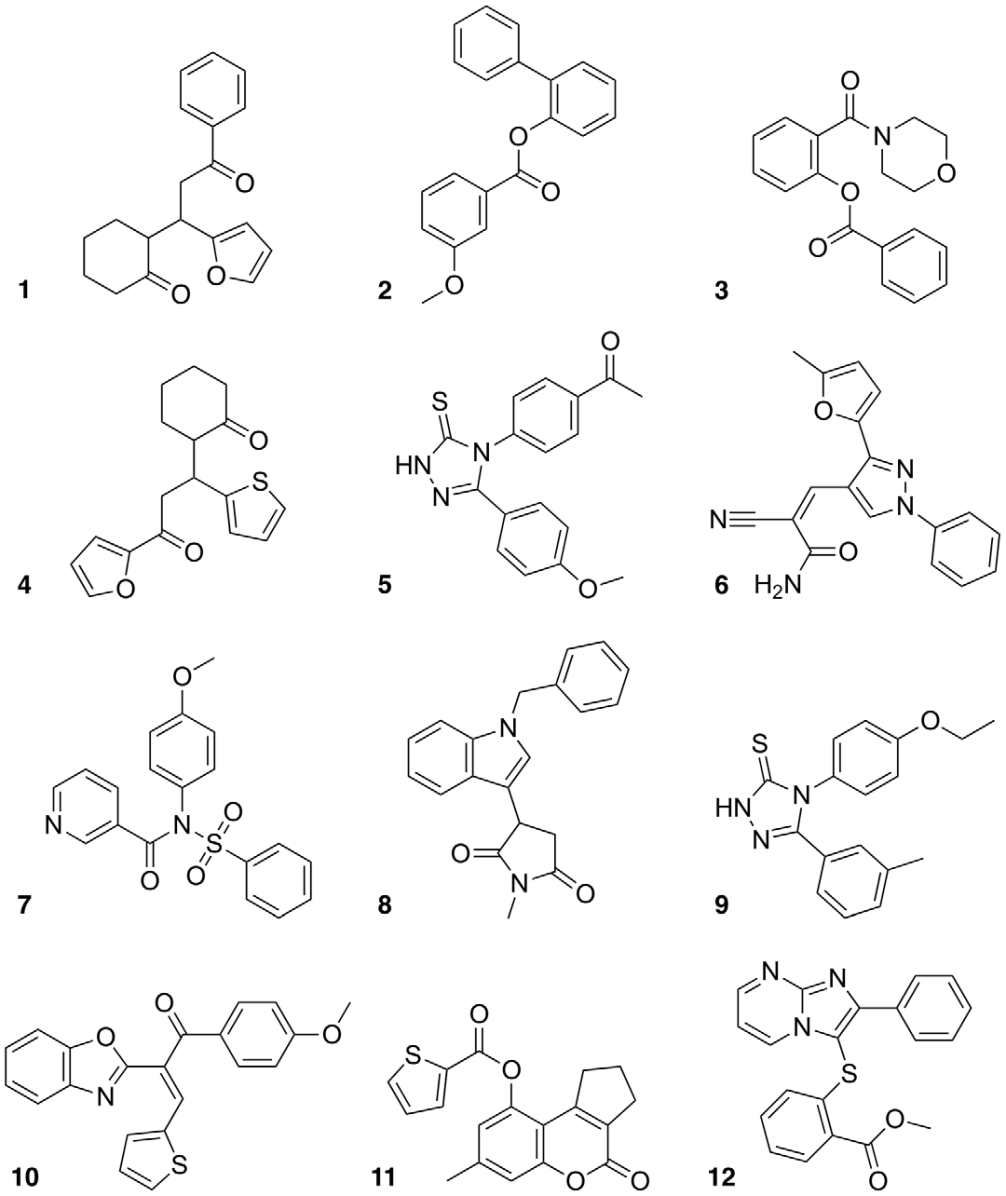
Compounds selected for the COX inhibition assay.

In the second virtual screening step, SpH was used for shape-based filtering. Two reference molecules (SC-558 and indomethacin; Fig. 4) resulted in two ranked lists of the pre-filtered ChemBridge compounds. 10 duplicates were found among the 50 top-ranking compounds from the two lists (20% overlap). In total, 12 compounds were selected by visual inspection, preferring potentially new scaffolds (‘cherry-picking’, Fig. 7), and submitted for activity determination in a direct enzyme inhibition and a whole blood assay.

### Assay results

**Figure 8:**
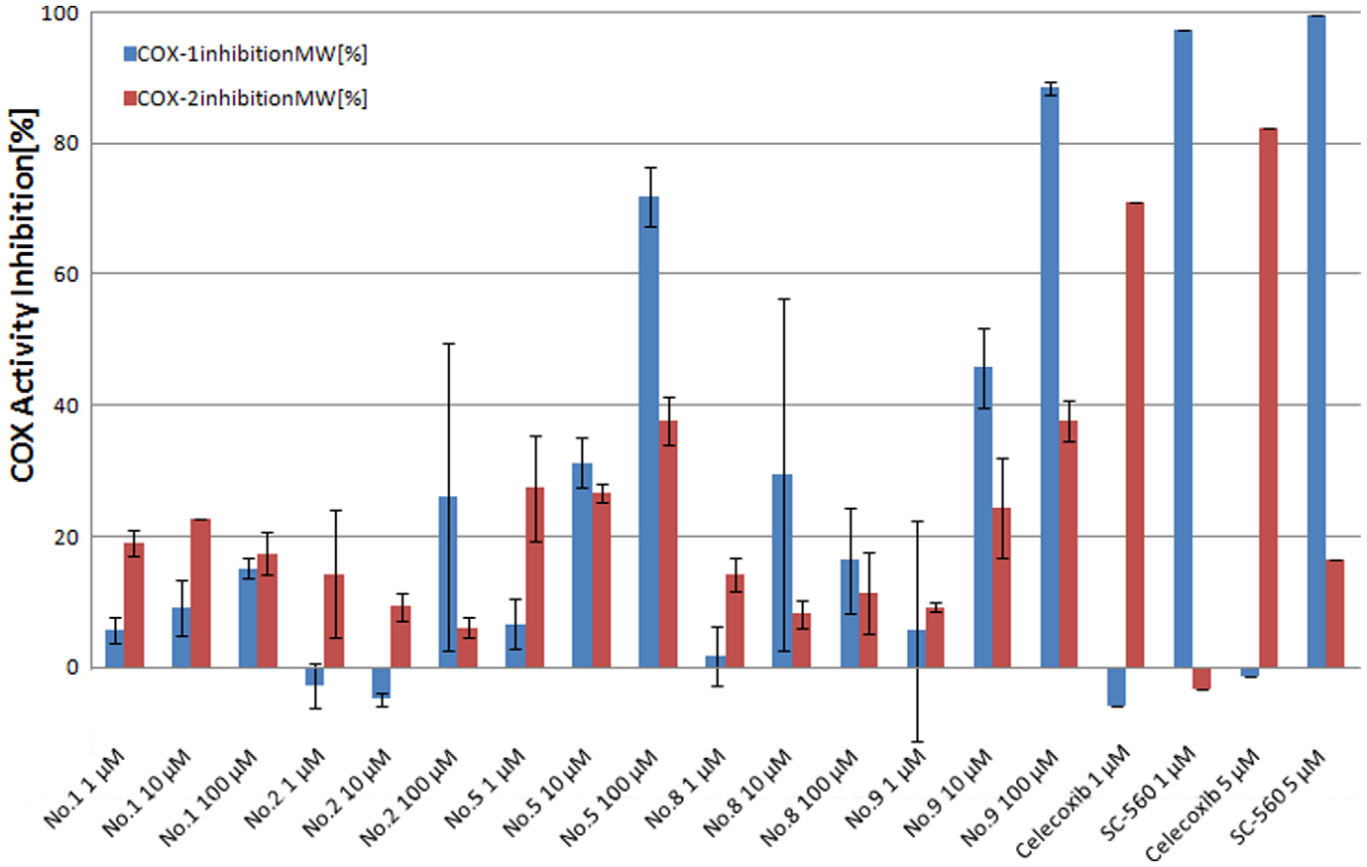
COX-2 inhibition *in vitro* assay results. Shown are COX-1 (blue) and COX-2 (red) inhibition. Celecoxib and SC-560 are known inhibitors selective for COX-2 and COX-1, respectively.

We determined the COX-inhibitory activity of 12 compounds by performing a commercially available competitive COX-inhibition assay using purified COX-1 (ovine) and COX-2 (human recombinant) enzymes. Compounds **5** and **9** inhibit COX-1 in a concentration dependent manner (Fig. 8 and Table 2). At 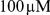 compounds **5** and **9** inhibit COX-1 activity to 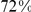 and 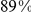, respectively. Both compounds have only marginal effects on COX-2-activity at concentrations up to 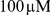. All other substances have no effect on COX-1 or COX-2 activity in this *in vitro* assay. While this outcome supports our general virtual screening approach, we failed to retrieve COX-2 inhibitors. This might be a consequence of using the selective COX-2 inhibitor SC-558 in combination with the non-selective COX inhibitor indomethacin as queries for the spherical harmonics shape filter. Apparently, the COX activity island on the SOM and SpH consensus filtering eliminated COX-2 specific features. It is also possible that there were no hitherto unidentified COX-2 ligands in the compound pool.

**Table 2:** Results of in vitro enzyme inhibition assay tests.

	COX-1 [%]						COX-2 [%]					
ID	1 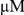		10 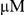		100 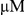		1 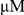		10 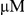		100 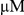	
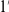	6±	2.0	9±	4.2	15±	1.6	19±	2.1	23±	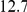 †	17±	3.2
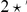	−3±	3.5	−5±	1.0	26±	23.5	14±	9.7	9±	2.2	6±	1.6
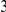	8±	1.3	12±	3.5	10±	5.1	19±	0.7	10±	1.9	14±	0.1
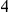	6±	10.7	−6±	3.8	−2±	2.8	3±	3.2	3±	7.4	−1±	9.9
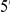	**7**±	**3.9**	**31**±	**3.8**	**72**±	**4.5**	**27**±	**8.1**	**27**±	**1.4**	**38**±	**3.7**
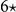	16±	4.7	18±	9.3	15±	1.6	19±	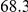 †	0±	10.0	12±	2.9
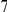	2±	4.5	4±	8.4	7±	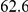 †	20±	9.4	13±	8.8	15±	0.3
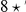	2±	4.5	29±	26.8	16±	8.0	14±	2.6	8±	2.1	11±	6.2
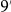	**6**±	**16.8**	**46**±	**6.1**	**89**±	**1.0**	**9**±	**0.7**	**24**±	**7.6**	**38**±	**3.1**
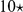	−7±	1.6	−10±	8.5	7±	2.8	2±	5.5	−2±	2.7	−1±	3.7
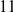	11±	12.6	5±	0.6	1±	1.5	5±	2.9	4±	4.2	0±	1.1
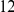	18±	4.0	8±	4.5	14±	1.5	15±	0.1	15±	7.5	19±	2.2

**Figure 9:**
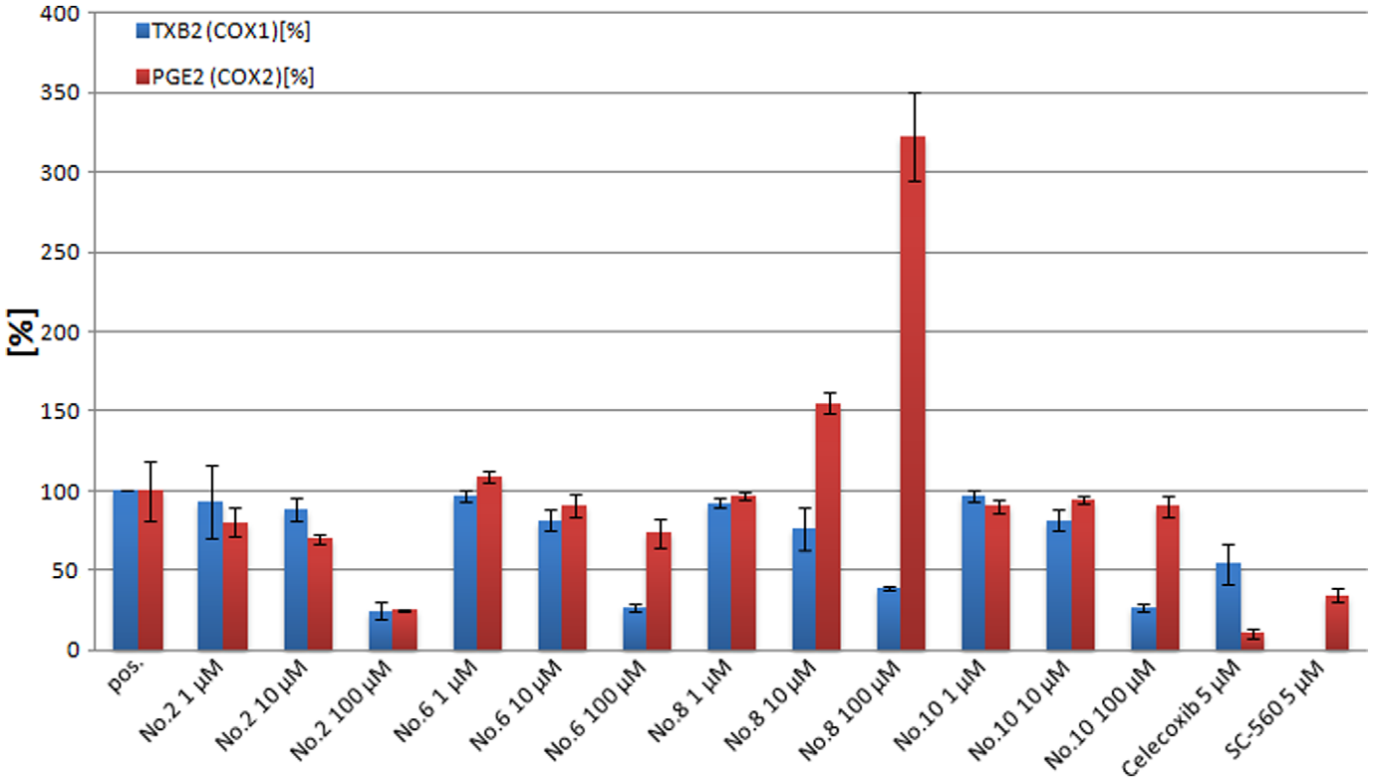
COX-2 inhibition whole blood assay results. Shown are 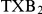 (blue, indicative of COX-1 activity) and 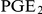 (red, indicative of COX-2 activity) amounts relative to the control (DMSO). Celecoxib and SC-560 are known inhibitors selective for COX-2 and COX-1, respectively.

Primed (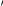) compounds are shown in Fig. 8, starred (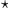) compounds are shown in Fig. 9. Discussed compounds are shown in bold face.

† For these three measurements, high standard deviations were observed. This could be due to solubility problems, impurities, protein degradation, or other unspecific effects. The corresponding measurements should be treated carefully.

In the whole blood assay (Fig. 9, Table 3), compounds **5** and **9** are less effective, with maximum COX-1 inhibition of about 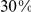 and no COX-2 inhibitory efficacy. Interestingly, in this assay, compounds **6**, **10**, **2** and **8** inhibit 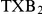 production in a concentration dependent manner up to 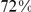, 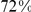 and 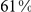 at 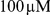, respectively. Compounds **6** and **10** have only marginal inhibitory potency on 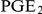 production, which points to selective COX-1 inhibitors *in vivo*. Compound **2** also inhibits 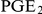 production comparable to 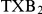, indicating that this compound is a COX-unselective inhibitor. In contrast, substance **8** increases the 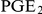 amount in a concentration dependent manner, which argues for an activator of 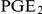 production in the cellular context. All other compounds show only very weak or no effect on 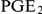 production.

**Table 3:** Results of whole blood assay tests.

	TXB 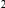 [%]						PGE 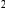 [%]					
ID	1 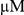		10 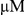		100 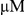		1 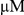		10 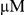		100 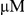	
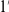	122±	15.0	94±	3.4	81±	1.2	105±	4.9	112±	8.4	97±	19.9
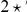	**93**±	**22.7**	**88**±	**6.9**	**25**±	**5.3**	**80**±	**9.0**	**70**±	**3.1**	**25**±	**0.5**
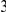	107±	0.0	107±	2.8	91±	2.3	93±	15.8	91±	8.9	88±	34.5
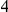	102±	3.5	91±	4.4	85±	1.8	110±	12.3	111±	16.5	105±	22.0
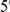	100±	0.9	98±	1.3	71±	1.3	105±	7.9	118±	6.9	106±	33.8
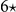	**97**±	**3.8**	**82**±	**6.6**	**28**±	**2.4**	**109**±	**3.6**	**91**±	**7.4**	**74**±	**9.1**
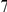	102±	6.3	89±	12.1	77±	5.8	99±	13.7	86±	11.9	93±	21.2
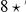	**93**±	**2.8**	**77**±	**13.3**	**39**±	**1.2**	**97**±	**2.5**	**155**±	**6.7**	**322**±	**27.5**
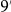	86±	6.9	71±	2.3	67±	12.1	73±	6.1	84±	9.8	89±	3.1
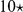	**97**±	**3.8**	**82**±	**6.6**	**28**±	**2.4**	**91**±	**4.2**	**94**±	**2.9**	**90**±	**6.3**
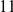	92±	1.8	87±	2.7	60±	1.9	105±	2.1	98±	9.2	69±	6.7
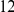	100±	1.3	85±	4.4	80±	0.9	102±	10.6	120±	9.4	113±	14.5

TXB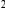 is indicative of COX-1 activity, PGE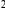 of COX-2 activity relative to DMSO control. Primed (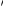) compounds are shown in Fig. 8, starred (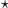) compounds are shown in Fig. 9. Discussed compounds are shown in bold face.

The inhibitory data obtained from the whole blood assay might be meaningful for further hit optimization. Compounds that are active in this assay are not snatched away by binding to serum albumin, but cross the cell membrane and overcome possible interactions with cellular substances or enzymes. This could explain why compounds **5** and **9** are active in the enzyme assay, but inactive in the whole blood assay. In contrast, compounds **6**, **10**, **2** and **8**, which were more active in the whole blood assay, possibly interact with the arachidonic acid pathway in other ways than direct inhibition of COX-1 or COX-2. Also, these compounds might be metabolized by cellular enzymes to more active derivatives, but this hypothesis needs to be tested by further experiments. Compound **8** is of special interest, as it induces 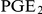 production up to 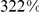. This increase could be due to an activation of enzyme activity, possibly by binding to the “inactive” monomer of the COX-homodimer complex [73], [74], or, due to an enhancement of COX-2 protein, either by transcriptional or post-transcriptional mechanisms.

As a preliminary novelty check, similarity searches were performed using SciFinder Web (2010-10-21) for data retrieval from the CAS database (Chemical Abstracts Service, Columbus, Ohio, USA; www.cas.org). For none of the actives any reference to COX inhibition was found, and only for compound **9** substructure matches (lacking the *meta* methyl group) were retrieved with regard to bioactivities other than COX inhibition. It is therefore reasonable to conclude that COX inhibition by compounds **5** and **9** represents a novel finding resulting from our study. We did not perform additional analytical investigations of compound integrity and purity other than those provided by the compound supplier. Therefore, we cannot exclude that the activities measured in the assays might be partially owed to decomposition or oxidation products. Analog compound design and testing will be mandatory.

### Conclusions

We presented a favorable retrospective evaluation of the SpH approach using COX-2 data from the DUD collection, and in a first prospective application demonstrated the usefulness of the descriptor in combination with a self-organizing map for retrieving bioactive ligands from a large compound pool. Although we did not retrieve a potent COX-2 inhibitor, which is likely owed to the setup of the virtual screening cascade, two novel COX-1 inhibitors were discovered. Future research will have to focus on mathematical descriptions of molecular shape that allow for enzyme subtype-selective ligand screening.

We introduced the magnitude of spherical harmonics coefficients as a partially rotation-invariant descriptor of molecular shape. In retrospective validation on the DUD dataset, the performance (as estimated by ROC AUC) of our shape-only method was comparable to other shape-based similarity searching methods. Results show that the magnitude of spherical harmonics decomposition coefficients can be used to describe molecular shape in a partially rotation-invariant way, resulting in a notable enrichment of active compounds in virtual and real screening studies. The combination of pharmacophore filtering by a self-organizing map and shape-filtering by spherical harmonics descriptors might be a useful two-step virtual screening protocol for hit retrieval from large screening compound collections.
